# Ethical Considerations in Fetal Cardiology

**DOI:** 10.3390/jcdd11060172

**Published:** 2024-06-01

**Authors:** Stefani Samples, Rupali Gandhi, Joyce Woo, Angira Patel

**Affiliations:** 1 Division of Pediatric Cardiology, Ann & Robert H. Lurie Children’s Hospital of Chicago, Northwestern University Feinberg School of Medicine, Chicago, IL 60611, USA; 2 Division of Pediatric Cardiology, Advocate Christ Children’s Hospital, Oak Lawn, IL 60453, USA; 3 Section of Cardiology, Department of Pediatrics, Comer Children’s Hospital, University of Chicago, Chicago, IL 60637, USA

**Keywords:** fetal cardiology, fetal echocardiogram, ethics, shared decision-making, innovation

## Abstract

Fetal cardiology has evolved over the last 40 years and changed the timing of diagnosis and counseling of congenital heart disease, decision-making, planning for treatment at birth, and predicting future surgery from the postnatal to the prenatal period. Ethical issues in fetal cardiology transect multiple aspects of biomedical ethics including improvement in prenatal detection and diagnostic capabilities, access to equitable comprehensive care that preserves a pregnant person’s right to make decisions, access to all reproductive options, informed consent, complexity in shared decision-making, and appropriate use of fetal cardiac interventions. This paper first reviews the literature and then provides an ethical analysis of accurate and timely diagnosis, equitable delivery of care, prenatal counseling and shared decision-making, and innovation through in utero intervention.

## 1. Introduction

Fetal cardiology as a subfield of pediatric cardiology has evolved over the last 40 years and changed the timing of congenital heart disease (CHD) diagnosis [1]. Through technical development and sonographer and physician education, fetal echocardiography has become a highly sensitive and specific noninvasive tool for detection, classification, and risk assessment of fetal cardiovascular disease [2]. Diagnosis of CHD in utero has shifted the focus of counseling, decision-making, planning for prompt treatment at birth, and predicting future management and outcomes to the prenatal period [3]. Additionally, the field is increasingly multidisciplinary with pediatric cardiologists, radiologists, obstetricians, maternal fetal medicine specialists, and neonatologists collaborating to care for the pregnant person and the fetus with the common goal of improving outcomes for CHD [4]. With rapid evolvement, ethical considerations emerge to maintain best practices and provide excellent cardiovascular care during the perinatal period, while supporting the pregnant person (and partner) through shared decision-making. In this paper, we review the existing literature and then provide an ethical analysis of accurate and timely diagnosis, equitable care delivery, prenatal counseling and shared decision-making, and innovation through in utero intervention.

## 2. Ethics in Fetal Cardiology

One of the most applied ethical frameworks in clinical practice, particularly in the United States and Canada, is the principles approach. The four principles are:

(1) Respect for autonomy, which requires respect for the decision-making capacities of autonomous persons;
(2) Nonmaleficence, which requires not causing harm to others;
(3) Beneficence, which is a group of principles requiring that we prevent harm, provide benefits and balance benefits against risks and costs;
(4) Justice, which is another group of principles requiring appropriate distribution of benefits, risks and costs fairly [5].

All four key principles should be considered and upheld in determining just action and resolving clinical ethical dilemmas. Performing an ethical analysis should not be viewed as creating a hierarchy of principles or choosing just one principle in a clinical situation. Rather, the four principles should be upheld simultaneously and equally when able. When this is impossible or there is direct conflict, consideration for how to mitigate the impact of the principles upheld less fully should be undertaken.

In fetal cardiology, the principle of respect for autotomy requires that physicians ensure informed consent from the pregnant person for continued or future treatment of the fetus. Informed consent generally consists of providing the patient with information about risks, benefits and alternatives that a “reasonable” person would want to know. The patient must have the capacity to make the decision, understand the relevant information that was provided, and must give their consent voluntarily (i.e., without coercion) [6,7].

Once the components of informed consent are established, the focus shifts to shared decision-making. Shared decision-making is a collaborative process that allows patients and clinicians to make healthcare decisions together, considers the best scientific evidence available, and incorporates the patient’s values, goals, and preferences. In fetal cardiology, shared decision-making should occur with members of the multidisciplinary team and the pregnant person. Shared decision-making is widely accepted as standard of care in the United States, Canada, Europe, and Australia [8]. Shared decision-making includes information exchange, deliberation, and ultimately decision-making by the pregnant person. Insisting on a course of action without considering family input or offering a dispassionate “menu” of possibilities does not respect patient autonomy, the role of the partner or other family members in the patient’s life, and the responsibility of the physician to provide direction. The medical team, guided by the principle of beneficence and nonmaleficence, adjusts recommendations accordingly. Most often, shared decision-making will require a series of conversations. Providing consistency in medical providers can help to limit confusion and mixed messaging. Despite widespread acceptance of this model, challenges still exist [9]. These include the patient’s goals, values, and preferences not being clear or easily communicated, diseases with little evidence-based data regarding risks or prognosis, cultural differences that lead to alternative understanding of health and disease, and varied preferences for degree of participation and control. These challenges are especially prevalent in fetal cardiology, especially as the diagnosis of a serious CHD can lead to emotional turmoil that may impact how the pregnant person and families assimilate the information that is being provided and act on that to make a decision. Finally, the principle of justice should guide the field as a whole to reduce disparities at all levels and promote equitable access to care.

## 3. Diagnosis and Delivery of Care

Diagnosis of CHD is the critical first step in the care of these medically complex patients. With advances in medical imaging, diagnosis is increasingly occurring prenatally, especially for critical CHD which often requires intervention early in life. However, despite improvements in fetal echocardiography, overall diagnostic rates of CHD remain relatively low, ranging from 36% to as high as 71% in some studies [10,11]. Prenatal diagnosis depends upon identification of risk factors by the primary obstetrician and/or identification of an abnormality on a screening ultrasound. CHD is more likely to be identified on screening ultrasounds when they are performed in hospitals or at maternal–fetal medicine practice offices where sonographers are more highly trained to identify cardiac abnormalities or there are larger practices with improved access to second opinions on acquired images [12]. With appropriate training and experience, sonographers can readily identify up to 70% of significant CHD on their own, showing that appropriate training could play a significant role in easing the burden of low prenatal diagnostic rates [13]. Part of this training includes improved outflow tract views, a recommendation made by the American Institute of Ultrasound for all second-trimester ultrasounds in 2013. However, appropriate identification of this view and any associated abnormalities continues to be challenging [12,14,15]. Within the general medical community, separate practice silos inherently restrict access to trained and experienced sonographers and physicians in most cases. Improving access would allow primary obstetricians to obtain feedback and outcomes from cases, thereby offering additional education to improve future screening ultrasounds [16]. Machine learning may also one day help improve the evaluation of fetal cardiac structures by optimizing image acquisition and quantification, thereby improving prenatal diagnosis capabilities [17]. However, machine learning has yet to demonstrate improved detection among patients with obesity, which is highly correlated with lower socioeconomic status [18]. As a result, the appropriate and equitable implementation of this is likely years away, given the need for rigorous training and validation of these methods.

Additionally, telemedicine may be a viable next option for expanding access to skilled providers. Videoconferencing can provide real-time feedback for sonographers during imaging as well as guidance for community physicians on care options and additional counseling availability for patients in complex cases. Several studies have already shown an impact of incorporating telemedicine on the care of pediatric cardiac patients and can provide the same additive value for fetal patients [19,20]. Virtual psychosocial interventions for fetal CHD patients and their partners have been studied as well and show promise for reducing distress and social isolation, as well as helping them feel more prepared and more hopeful [21]. Despite the potential improvements in access to care that prenatal telehealth may provide, we must ensure it does not exacerbate disparities, for instance, given unequal access to broadband internet [22].

Timely diagnosis of CHD (generally 18–20 weeks gestation) allows families to make management decisions in the context of their life and priorities. Urbanicity, potential financial burdens, religious beliefs, repercussions from family and community, and effects on other children can significantly impact their decision-making [23]. Many different types of providers can offer information on the various care options for families, including termination, palliative surgery, and hospice (“comfort care”) and the role of the primary obstetrician is especially important and relevant. Ethical consultations can additionally help families and providers navigate more complex cases, for example, when the family decides not to pursue standard surgical intervention and clinicians have ethical concerns about this choice. Providing information on all care options is particularly important when patients with lower education or socioeconomic level may preferentially defer to the medical teams’ recommendations [24].

Multiple studies have demonstrated inequities in prenatal detection of CHD based on social determinants of health. Particularly for those with public insurance, living in poorer neighborhoods, or rural communities, prenatal CHD diagnosis rates are lower [14,25]. The lack of universal screening contributes to this disparity between higher and lower socioeconomic patients [26]. Additionally, interventions for provider-, hospital-, and societal-level barriers to prenatal diagnosis can be researched and implemented—including facilitation of appointment scheduling and allowing time off work to obtain prenatal care without penalty—to directly mitigate these inequities [27].

Finally, the overturn of *Roe v. Wade* will disproportionately affect abortion access for pregnant individuals of color and low socioeconomic status, who, prior to the Dobbs decision, had higher rates of abortion than individuals of White race and higher socioeconomic status [28,29]. The Dobbs decision is also expected to increase the strain on the already overburdened healthcare system with an increase in children born with significant CHD [30]. A recent decision analysis predicted that a complete abortion ban would result in an approximate 50% increase in single ventricle cardiac defects, resulting in increased neonatal heart surgeries, heart transplants, and neonatal deaths due to CHD [31]. Additional demands on the medical work force and space constraints can further worsen disparities in access to care. One year after the Dobbs decision, there has been a documented decrease in abortions in states with the most restrictive laws, the largest of which occurred in states with complete bans, as expected [32]. Neighboring states without restrictive laws saw a comparable increase in their abortion rates, which subsequently led to an essentially unchanged national abortion rate [33]. The current American medical landscape demonstrates how integral it is to allow physicians, no matter where they practice, to provide the best evidence-based care for patients without significant legislative interference. The negative impacts of restricted abortion access have been previously studied, and Green et al. showed those without access to abortion had higher odds of poverty 6 months after delivery and this trend continued even 4 years later [34]. Given that abortion restrictions more severely affect low-income women, particularly those from marginalized racial and ethnic groups that already struggle with barriers accessing healthcare, these disparities would only be expected to grow over time [32].

## 4. Prenatal Counseling and Shared Decision-Making

Counseling after fetal diagnosis of CHD requires significant expertise given the variety and complexity of lesions that can be encountered. While research evaluating effective counseling has been limited, Kovacevic and colleagues have reported successful counseling occurring 44.7% to 46.3% of the time [35,36]. Domains of counseling rooted in the principles of nonmaleficence and beneficence include appropriate transfer of medical knowledge, trust in medical staff, transparency of treatment process, parental coping resources, and perceived situational control. Parents generally report desiring more information during counseling including information on quality of life, ongoing cardiology follow up, and certainty about how to describe their child’s heart disease to another medical professional [37]. Parents also note that feeling supported by physicians and having information relayed in easy-to-understand terms is important in their counseling sessions [9,38]. Parents value appropriate systems structure in their care including separate counseling rooms, access to a specialist soon after diagnosis, internet and social media resources, and patient navigators for additional support during pregnancy [9,34,39,40]. When compared to counseling before the COVID-19 pandemic, parents now report an increased need for many of these support mechanisms, particularly written and online information, given the increased anxiety and uncertainty surrounding medical outcomes that many families have experienced with the pandemic [41]. Parents report interpreting counseling negatively when feeling pressured to terminate the pregnancy by the physician’s counseling and were more likely to obtain a second opinion [42,43].

Physicians similarly report barriers to effectively completing successful counseling. While most physicians report not adapting counseling on complex issues such as termination of pregnancy and palliative care based upon their personal beliefs, these sensitive topics are not always routinely discussed in counseling sessions, even for the most complex CHD lesions such as hypoplastic left heart syndrome (HLHS) [44,45]. When there is no gold standard for treatment, physicians also report not always discussing nonsurgical management, transplant potentials, and other options which may not be available at their own institution [46]. In internet surveys, physicians acknowledged the benefits from more formal training on counseling [44]. In similar survey-based research, less than 50% of physicians report having formal training and feedback on fetal counseling [47].

Given these differences in perception surrounding fetal counseling, appropriate communication can be challenging. Shared decision-making is recommended given the difficult choices for care pathways for fetal patients with significant CHD. Shared decision-making can be further facilitated by decision aids for families [48]. As the complexity of counseling can differ from case to case, each session must be tailored to the case complexity and individual family’s needs. A structured counseling session divided into two parts to allow transfer of medical information followed by questions from the families with a break in between appears to be the best approach [49].

When counseling families about fetal CHD, physicians must keep abreast of the current trends in outcomes from CHD surgical interventions, including the likelihood of mortality and common morbidities at one’s own institution and nationally. This includes considering the impact of additional extracardiac anomalies and genetic defects on these outcomes. Providing the most accurate information allows families to consider all potential outcomes when deciding on care pathways. There has been significant work on standardizing the data collection and reporting of outcomes for CHD, including adopting common nomenclature and a uniform dataset and mechanism of evaluating case complexity with collaboration between medical and surgical subspecialties [50]. Recent multi-specialty collaboration has put forth specific recommendations for both essential care and comprehensive care centers to better establish the basic requirements for centers caring for patients with CHD [51]. These detailed recommendations demonstrate the complexity of the care required for CHD patients and may aid fetal cardiologists to make better recommendations on delivery planning and care center expectations.

Alternatively, hospice and palliative care options (to include the broader definition of support for complex decision-making) has been gaining attention in recent years, though research on this topic still lags behind palliative care incorporation in other pediatric subspecialties [52]. Utilizing the previous learnings from palliative care research can help fetal cardiologists improve their counseling for CHD families, especially given the uncertainty which exists in predicting postnatal outcomes accurately [39,53]. In counseling patients regarding any management—termination, surgical intervention, or hospice—it is critical to recognize the educational, religious, cultural, and ethnic backgrounds of the pregnant individual and their family [49].

## 5. Innovation and Fetal Intervention

Fetal cardiac intervention (FCI) is an innovative therapy where a cardiac interventionalist accesses the fetus through the maternal abdomen, punctures the fetal heart, and performs a cardiac angioplasty intervention. The most common example of fetal cardiac intervention (FCI) is fetal aortic valvuloplasty. Based on the observation that severe fetal aortic stenosis at 20 weeks gestation progressed to HLHS by 32 weeks, the hypothesis was that fetal aortic valvuloplasty would improve flow through the left ventricle and aortic valve, and potentially allow the left ventricle to grow [54]. While the upfront risks are considerable (fetal demise), the long-term outcome was potentially a two-ventricle repair and, therefore, the benefit was thought to be worth the risk, if successful.

The first FCI was successfully performed in 2000 in pregnant persons carrying a fetus with HLHS [55]. Prior to this, in utero aortic valvuloplasty had been attempted in 12 reported cases by different interventionalists at later gestational ages. Of these cases, seven were technically successful but four of these fetuses died within 24 h of the procedure, and two died shortly after birth [56]. The last 25 years have brought advances in the techniques, resulting in improved technical success and survival. However, in nearly all fetuses thus far, FCI is the first procedure with additional postnatal procedures needed after birth and into childhood. FCI can be performed for evolving HLHS with fetal aortic valvuloplasty, for HLHS with restrictive atrial septum with a balloon atrial septostomy with or without stent placement, rarely as pulmonary valvuloplasty for critical pulmonary stenosis or developing pulmonary atresia with intact ventricular septum, and has also been reported once for transposition of the great arteries with an aneurysmal, immobile intact atrial septum [57].

As the earliest application of this technology, aortic valvuloplasty for developing HLHS is the most common type of FCI. Studies from the US and Europe have reported technical success of the fetal aortic valvuloplasty between 73 and 94% (improving as expected with later cohorts), with 36–59% of patients achieving a biventricular repair. The largest centers reported 55–59% of patients achieving a biventricular repair, highlighting the potential successes when highly specialized centers develop expertise in these rare and complex procedures. Selecting the optimal patients for FCI continues to be an evolving area potentially leading to improved outcomes [57,58,59,60].

## 6. Discussion

### 6.1. Ethical Framework for Fetal Diagnosis and Counseling

Ensuring accurate diagnosis, delivering the best care possible, and optimizing access can be viewed through the ethical principle of justice. There are several barriers to maximizing these areas of care and promoting justice. In a large multi-center study on HLHS and transposition of the great arteries specifically, lower socioeconomic status was associated with lower prenatal detection rates as well as later gestational age at time of prenatal diagnosis [61]. The same study also noted these disparities in association with rural residence and use of public insurance. Patients have similarly reported significant burden to prenatal diagnosis including social barriers such as difficulty with appointment scheduling, lack of transportation or having a significant distance to travel for care, and difficulty having time away from work for appointments [27]. There is concern that these disparate trends may continue as cardiac care becomes increasingly complex, with recommendations for significant resources needed for comprehensive care, which naturally will lead to regionalization of CHD care [51,62].

Fulfilling the ethical principles of respect for patient autonomy, beneficence and nonmaleficence, comprehensive counseling from fetal cardiologists, who are often the first point of contact for patients, should be as detailed as possible. Counseling includes information sharing of short- and long-term medical prognosis, risks to the pregnant person and fetus, and the impact of the diagnosis on the family unit. Fetal cardiologists educate the pregnant person and family unit to allow them to make a fully informed decision for themselves regarding the continuation of the pregnancy as well as peripartum and postnatal care.

The ability to fully counsel patients has been significantly curtailed by the recent Supreme Court decision *Dobbs v. Jackson*, which overturned *Roe v. Wade* and led to restricted reproductive rights in many states. Multiple professional societies have issued statements commenting on the impact of this decision. The American College of Obstetrics and Gynecology (ACOG) calls it an “Infringement on respect for patient autonomy and the sanctuary of physician-patient relationship… this decision is a direct blow to bodily autonomy, reproductive health, patient safety, and health equity in the United States. The principle of shared decision-making is founded on respect for peoples’ expertise in their own bodies and lives and clinicians’ expertise in science and medicine” [63]. In addition, ACOG states that medical physical harm will occur because pregnancy-related health concerns can become worse, there is morbidity and mortality associated with childbirth, and restricting access to abortion will increase inequities in medical care.

The ACOG statement essentially rephrases the underlying framework of bioethical principles that physicians are obligated to uphold: respect for autonomy, beneficence, nonmaleficence, and justice. Professional societies that care for pregnant persons and fetuses have stated the following: “It is our obligation not only to educate the expectant patient and family regarding the fetal condition, but also to allow them the opportunity to make a fully informed decision regarding pregnancy, peripartum and postnatal care, for both themselves and their fetus” [64]. In fetal and pediatric cardiology, multiple societies are aligned on how Dobbs impacts full counseling (infringement on physician duty to offer unbiased counseling and all reasonable medical management options to pregnant persons) and access to reproductive health, and there is risk for significant harm (e.g., to pregnant person themselves in cases where pregnancy itself is life-threatening, limits on identification of terminal or nonviable pregnancy, risk to other fetus in twin pregnancy) [65].

Even when there are no legal barriers to counsel fully, an area of potential ethical dilemma during the process of shared decision-making is determining what diseases to acknowledge the options of termination of the pregnancy or comfort care after delivery [43,45]. Prenatally, one may be unlikely to mention the option of termination of pregnancy if the fetal diagnosis is a simple ventricular septal defect, but if the fetus has hypoplastic left heart disease or a complex heterotaxy where mortality and morbidity outcomes are improved but still high, a discussion of pregnancy termination may be important. Additionally, the quality of parental counseling can vary depending on availability of multi-disciplinary sessions attended by various provider groups such as fetal cardiologists, neonatologists, obstetrics, maternal fetal medicine, cardiovascular surgeon, genetics, and palliative care.

Additionally, whether there is ever the possibility of “true” informed consent and understanding during the process of shared decision-making can be challenging in fetal cardiology and CHD. Based on the complexity of CHD, outcomes can vary significantly, there is real ambiguity especially in the context of other comorbidities, and current outcomes do not account for innovation in treatments and surgeries. When counseling a pregnant person about the fetal cardiac disease, it has not always been clear how to share the unknown and the known future treatments and/or problems that the child could have. For example, when counseling a pregnant person and partner about fetal hypoplastic left heart disease, some may choose to counsel about rare—but significant—long-term complications such as plastic bronchitis and protein-losing enteropathy, where other fetal cardiologists leave these complications as vague possibilities or do not mention them at all. A physician may be biased based on his or her own experience, data show that uniformity in counseling is lacking, and there is variation in how much information to discuss [44,66]. However, recent evidence does suggest that most parents prefer to have more information that may provide guidance for the future [36,67,68]. Future research is needed to improve this process of shared decision-making, develop standards and best practices, and determine how to provide information to parents in a way that is helpful, does not increase stress and anxiety, and does not translate to a worse-case scenario conversation [49].

### 6.2. Ethical Framework for Determining Whether Fetal Catheter Interventions Are Acceptable

Innovative procedures to treat CHD have advanced the field considerably in a short period of time. Procedures such as the Blalock–Thomas–Taussig shunt and development of ECMO are just some examples. These procedures, when developed, were usually considered ethically reasonable because the alternative without the treatment was death or severe morbidity. The focus, therefore, was on ensuring that the parent/decision-maker understood the risks of the procedure, and the risks of not going forward with it [69]. Fetal cardiac interventions for HLHS may fall into this category but some could argue that the survival with a three-staged palliative surgical approach at a high-volume comprehensive center with good reported outcomes makes the option for FCI less desirable.

The field of ethics has also progressed over the years, and complete transparency about projected risks and benefits is expected from patients. Although desirable goals, shared decision-making coupled with the goal of transparency may make it difficult for innovative procedures to move forward at the same rate that they progressed in the past [70]. As surgical techniques and outcomes have improved, those promoting innovative procedures must consider ethical concerns because many of the current practices have relatively good success and long-term survival. How can one decide whether the chance at incremental improvement in the technique and outcome, which might benefit an entire group of patients with a particular disease, is worth the upfront risk incurred by one single patient?

When the risks and benefits are discussed with the pregnant person and partner, FCI should be presented as the first step of multiple procedures and surgeries. Traditionally, only the specific medical improvement to the fetus was weighed as a benefit and the risks included all the possible morbidities and mortalities to the fetus and the pregnant person. A more progressive way of weighing the benefits and risks allows the evidence-based psychosocial benefits of fetal therapy to be considered, i.e., the benefit of having a baby with decreased disease burden or a longer lifespan, or the sociological benefit of the pregnant person feeling that they have taken action to help their child [71]. This more cohesive approach has been lauded as a new framework for the ethics of fetal interventions and is one that captures more of the true essence of why fetal therapies are sought out by pregnant persons and their partners [72]. Furthermore, limiting fetal therapies to only life-threatening conditions unnecessarily restricts these therapies. Instead, the risks to the fetus and pregnant person should be assessed against the potential benefits to the fetus, pregnant person, and the study’s societal value [72].

Fetal interventions have the added complexity of balancing the benefit to the fetus and mother with the potential harms to both. It is important to recognize that those on the medical treatment team may not assess these maternal and fetal risks and benefits similarly. Antiel et al. analyzed the relative importance of social and ethical concerns among MFM physicians, pediatric surgeons and neonatologists. They surveyed these three groups of physicians on the relative importance of nine different considerations, some of which were the extent to which the future child benefits from the operation, the risk of fetal demise, and the risk of maternal complications. They then identified four physician groups based on the content of what they rated as most important to their decision. They labeled these as fetocentric, risk-sensitive, maternal autonomy, and family impact/social support. They found that all physicians ranked neonatal benefit and risk of maternal complications as highly important; however, they ranked secondary areas differently. Although there was considerable overlap between preferences, they found that surgeons were more likely to align with the risk-sensitive group, neonatologists the fetocentric group, and MFMs with family impact/social supports [73]. The authors conclude that understanding these nuanced preferences may help manage difference of opinions on how to best care for the patient and fetus.

Finally, when discussing innovation, one must consider that the learning curve with any new technique or operator often means an initial increase in morbidity and mortality. For example, the group at Boston Children’s Hospital have developed an FCI program over the past 20 years. The learning curve was evidenced by the improvement in technical success to 94% in the later cohort (2009–2015) as compared to 73% in the early cohort (2000–2008) [56,58]. The latter era also had an increase in biventricular outcome (59% as compared to 26%) [58]. Although the overall case numbers of FCI may have increased over the years, the volume at any individual center remains low, and it is possible that the learning curve at low-volume centers may never advance. When developing an innovative procedure such as FCI, time must be spent assessing the historical knowledge, crafting a multidisciplinary team, and if possible, training the interventionalist at a center that has considerable experience [74,75]. It is an ethical imperative that FCI becomes regionalized without new centers launching in multiple geographically close areas. While this may create a care distance burden for the pregnant person, the higher likelihood of the FCI being technically successful at a high-volume center that has invested the time and created a multidisciplinary program outweighs the convenience of closer care at a low-volume center.

## 7. Future Directions

Future inquiry will need to focus on ensuring an equitable and just system that optimizes the care of the pregnant person and the fetus with CHD. The gaps include the following: improving overall detection rate and decreasing disparities in detection by region and socioeconomic status, refining counseling and shared decision-making, considering how referral for surgery after birth will evolve given the new definition of essential versus comprehensive care centers, understanding the impact of limitations on reproductive rights in fetal cardiology, and continuing to pursue innovative therapies in an ethical manner.

## 8. Conclusions

Ethical issues in fetal cardiology transect multiple aspects of biomedical ethics including improving prenatal detection and diagnostic capabilities, access to equitable comprehensive care that preserves pregnant person’s right to make decisions, access to all reproductive options, informed consent, complexity in shared decision-making, and appropriate use of fetal intervention. To maximize ethical, appropriate patient care, and to continue to evolve the field of fetal cardiology, the clinician needs a holistic understanding of the unique aspects of fetal cardiac care and the ethical issues that can emerge.
